# Complex Sequence‐Defined Heteropolymers Enable Controlled Film Growth in Layer‐By‐Layer Assembly

**DOI:** 10.1002/marc.202400482

**Published:** 2024-08-06

**Authors:** Ranajit Barman, Michel Tschopp, Laurence Charles, Gero Decher, Olivier Felix, Jean‐François Lutz

**Affiliations:** ^1^ Université de Strasbourg, CNRS, UMR 7006, ISIS Laboratory of Chemistry of Informational Macromolecules 8 allée Gaspard Monge Strasbourg 67000 France; ^2^ Université de Strasbourg, CNRS Institut Charles Sadron UPR22 23 rue du Loess, Strasbourg Cedex 2 67034 France; ^3^ Aix Marseille Université, CNRS, UMR 7273 Institute of Radical Chemistry, Marseille Cedex 20 13397 France

**Keywords:** data storage, digital polymers, layer‐by‐layer assembly, sequence‐controlled polymers, thin films

## Abstract

Digitally‐encoded poly(phosphodiesters) (*d*‐PPDE) with highly complex primary structures are evaluated for layer‐by‐layer (LbL) assembly. To be easily decoded by mass spectrometry (MS), these digital polymers contain many different monomers: 2 coding units allowing binary encryption, 1 cleavable spacer allowing controlled MS fragmentation, and 3 mass tags allowing fragment identification. These complex heteropolymers are therefore composed of 6 different motifs. Despite this strong sequence heterogeneity, it is found that they enable a highly controlled LbL film formation. For instance, a regular growth is observed when alternating the deposition of negatively‐charged *d*‐PPDE and positively‐charged poly(allyl amine hydrochloride) (PAH). Yet, in this approach, the interdistance between consecutive coded *d*‐PPDE layers remains relatively small, which may be an issue for data storage applications, especially for the selective decoding of the stored information. Using poly(sodium 4‐styrene sulfonate) (PSS) as an intermediate non‐coded polyanion, it is shown that a controlled interdistance between *d*‐PPDE layers can be easily achieved, while still maintaining a regular LbL growth. Last but not least, it is found in this work that *d*‐PPDE of relatively small molecular weight (i.e., significantly smaller than those of PAH and PSS) still enables a controlled LbL assembly.

## Graphical abstract

Nano‐organized thin films containing digital layers are prepared by layer‐by‐layer deposition of digitally‐encoded poly(phosphodiester)s with non‐coded polyelectrolytes such as poly(allyl amine hydrochloride) and poly(sodium 4‐styrene sulfonate). Despite the sequence heterogeneity of the digital polymer and the differences in molecular weight between coded and non‐coded polyelectrolytes, a linear film growth is observed, thus resulting in the formation of well‐segregated digital strata.

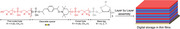

## 1 Introduction

Macromolecular information storage has recently emerged as an exciting new option for data storage.^[^ ^1^, ^2^, ^3^ ^]^ Inspired by natural information storage media such as chromosomes, it consists in storing information at the molecular level in a linear polymer chain using a controlled monomer sequence. For instance, digital information can be stored using a coded alphabet of at least two monomers,^[^ ^4^, ^5^ ^]^ thus opening new opportunities for applications in information technologies. Most of the work in the area has been dedicated to DNA,^[^ ^6^, ^7^ ^]^ which is an obvious choice because its natural function is to store data. Yet, it has been shown in recent years that macromolecular storage can also be achieved using synthetic polymers.^[^ ^8^, ^9^, ^10^, ^11^, ^12^ ^]^ The first example of such a non‐biological digital polymer was reported in this journal in 2014.^[^ ^13^ ^]^ Since then about twenty different families of digital polymers have been reported, as listed in recent reviews.^[^ ^3^, ^14^ ^]^ Overall, synthetic digital polymers offer interesting advantages over DNA because their properties can be tuned using a broad variety of building‐blocks and chemical reactions.^[^ ^15^ ^]^

Nevertheless, there is still an important technological gap between natural and non‐natural informational polymers. The main reason for that is that the synthesis, sequencing, and nanotechnology of DNA have been extensively studied over decades, whereas the field of synthetic digital polymers is still emerging. For instance, to process large amounts of data, libraries of synthetic digital polymers shall be organized in space (i.e., in 2D or 3D organized materials). This aspect is still underexplored,^[^ ^16^ ^]^ even though some strategies have been reported such as physical mixtures,^[^ ^17^, ^18^ ^]^ micro‐arrays,^[^ ^19^ ^]^ multi‐well plates,^[^ ^10^ ^]^ tubes set,^[^ ^20^ ^]^ crystals,^[^ ^21^ ^]^ self‐assembled constructs^[^ ^22^ ^]^ and structured thin films.^[^ ^23^ ^]^ In the latter case, we have recently shown that the layer‐by‐layer (LbL) deposition of polyelectrolytes^[^ ^24^, ^25^, ^26^ ^]^ is a promising approach for preparing digital multilayers. In that earlier work,^[^ ^23^ ^]^ we have synthesized a library of sixteen different digital poly(phosphodiester)s (*d*‐PPDE), which are polyanions. These negatively‐charged polymers were used for LbL assembly in conjunction with poly(allyl amine hydrochloride) (PAH) as a counter‐polycation and thin films were constructed using various deposition methods (i.e., manual dipping, robotic dipping, and spin‐assisted assembly). Interestingly, at each layer poly(phosphodiester)s containing different information sequences were deposited, thus allowing the formation of stratified thin films for data storage. Yet, in order to attain a perfect segregation of coded layers, the polymer pair *d*‐PPDE/PAH was not sufficient, and intermediate non‐coded layers had to be created using poly(sodium 4‐styrene sulfonate) (PSS) and PAH.^[^ ^23^ ^]^

In this previous work, *d*‐PPDE was synthesized using only two coded phosphoramidite monomers **0** and **1**. This basic binary alphabet was selected in order to prepare model polyelectrolytes and to study their viability for the formation of LbL digital films. However, such model *d*‐PPDE is not optimal for mass spectrometry sequencing. It has been reported that above a critical chain‐length, standard *d*‐PPDE cannot be decoded by MS.^[^ ^27^ ^]^ To bypass this issue, a more elaborated molecular design was reported in 2017.^[^ ^28^ ^]^ It relies on the use of alkoxyamine‐containing inter‐byte cleavable spacers that allow a controlled chain‐fragmentation and decoding by a pseudo‐MS^3^ strategy. This strategy also requires the use of byte tags, which permit to identification of the formed fragments. In recent years, the design of these polymers has been improved, and optimized molecular structures have been reported for both spacers and tags.^[^ ^29^, ^30^, ^31^ ^]^ Nevertheless, these optimal structures for sequencing are complex heteropolymers containing very different types of building‐blocks (e.g., coded units, spacers, and tags) as well as easily cleavable bonds (i.e., alkoxyamines). Complex heteropolymers made of many different comonomers have rarely been investigated for LbL film formation.^[^ ^32^ ^]^ In this context, the aim of the present work is to study the validity of complex *d*‐PPDE heteropolymers for LbL assembly.

## 2 Results and Discussion

**Figure 1:**
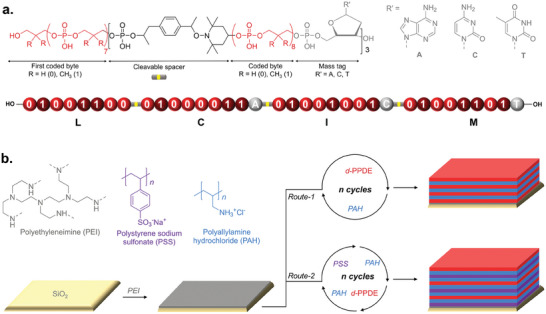
a) General molecular structure of the digital heteropolymer studied in this work. b) Molecular structures of non‐coded polyanion and polycations that were used in the construction of layer‐by‐layer (LbL). The digital multilayer film was prepared following two routes. Route 1: alternating deposition cycle of coded *d*‐PPDE (red) and non‐coded PAH (blue). Route 2: four‐step PSS/PAH/*d*‐PPDE/PAH deposition cycle.

In order to investigate the validity of complex heteropolymers in LbL assembly, a *d*‐PPDE (**Figure** **1**a) was first synthesized by solid‐phase phosphoramidite chemistry, using previously‐optimized protocols.^[^ ^33^, ^34^ ^]^ The acronym of the Laboratory of Chemistry of Informational Macromolecules (LCIM) was stored in the polymer using extended ASCII code. Each letter of the acronym corresponds to one byte, which is composed of a sequence of eight binary monomers containing either a propyl phosphate (**0**) or a 2,2‐dimethylpropyl phosphate (**1**) motif.^[^ ^33^ ^]^ Following a previously‐established convention,^[^ ^27^ ^]^ the reading direction of the ASCII‐encoded message is opposite to the synthesis direction. Besides the coded monomers, two other types of monomers were incorporated in the *d*‐PPDE chains (Figure S1, Supporting Information): an alkoxyamine‐containing rigid spacer (RISC2)^[^ ^29^ ^]^ that is periodically placed between bytes and mass tags (i.e., nucleoside phosphoramidites) that are included at the end of each byte except the first one.^[^ ^28^ ^]^ Thus, one *d*‐PPDE heteropolymer contains in total 38 monomers units including 32 binary monomers **0**/**1**, 3 RISC2 spacers, and 3 mass tags A, C, and T (Figure 1a). As evidenced in earlier work,^[^ ^29^ ^]^ the NO─C bonds of RISC2 selectively cleave in low collision induced dissociation (CID) MS/MS conditions and generate mass‐tagged intact bytes, which can afterward be individually fragmented and decoded by a pseudo‐MS^3^ strategy.^[^ ^35^ ^]^ The use of mass tags permits to identification of the original location of each byte in the *d*‐PPDE chain. Consequently, the complete coded sequence can be deciphered and reconstructed.

The structural uniformity of the formed polymer was evaluated by HPLC (Figure S2, Supporting Information) and electrospray mass spectrometry (ESI‐MS) (Figure S3, Supporting Information). Both techniques confirmed the formation of the sequence‐coded heteropolymer. In the negative MS mode, the targeted sequence was detected with charge states ranging from 7^−^ to 16^−^ (in green in Figure S3, Supporting Information), as supported by its successive fragmentation pattern obtained by MS/MS (Figure S4, Supporting Information) and MS^3^ (Figure S5, Supporting Information). A low abundance impurity was also observed (in grey in Figure S3, Supporting Information) with a mass difference Δm = −166.0 Da as compared to the targeted species. This signal corresponds to a small fraction of truncated *d*‐PPDE chains missing a **1** bit.

Layer‐by layer (LbL) assembly was then investigated using the synthesized *d*‐PPDE heteropolymer and PAH as a polyanion/polycation pair. At first, multilayers were constructed by simply alternating the deposition of these oppositely charged polyelectrolytes (route 1 in Figure 1b). The aim of this first approach was to check the bare influence of the primary structure of the heteropolymer on multilayer growth and on the morphology of the formed LbL film. For data storage applications, the digitally‐encoded layers shall be well separated from one another to facilitate the reading of the encoded information in nanostructured LbL films.^[^ ^23^ ^]^ Hence, exponential growth regimes that have already been observed with some polyphosphates shall be avoided.^[^ ^36^ ^]^ Indeed, in such materials, polyelectrolyte migration between layers occurs.^[^ ^37^ ^]^ Therefore, a controlled regular growth is preferred. The LbL films were built on polyethyleneimine‐modified silicon wafers by a manual dipping method where the wafer was immersed in a solution of a polyelectrolyte (either PAH or *d*‐PPDE) for 15 min and then washed (3 times dipping in Milli‐Q water for 2 min each) to remove excess and weakly attached polyelectrolytes. Film growth was followed by ellipsometry after the fabrication of each layer. Figure S6 (Supporting Information) shows the results obtained for the deposition of ten PAH/*d*‐PPDE layers (concentration of *d*‐PPDE = 0.02%) in Route 1. Interestingly, the film was found to grow homogenously and linearly with an overall thickness of 27 nm and a root‐mean‐square (RMS) roughness of 0.20 nm. This implies that the complex composition of the heteropolymer does not prevent linear film growth. It is also important to note that the chain‐length and the molecular weight of the studied heteropolymer are significantly lower than in our previous study employing model PPDE homopolymers and copolymers.^[^ ^23^ ^]^ Despite the significant molecular weight difference between the polyanion and the polycation, structured LbL films can still be efficiently assembled, which is interesting for the preparation of digital layers.

**Figure 2:**
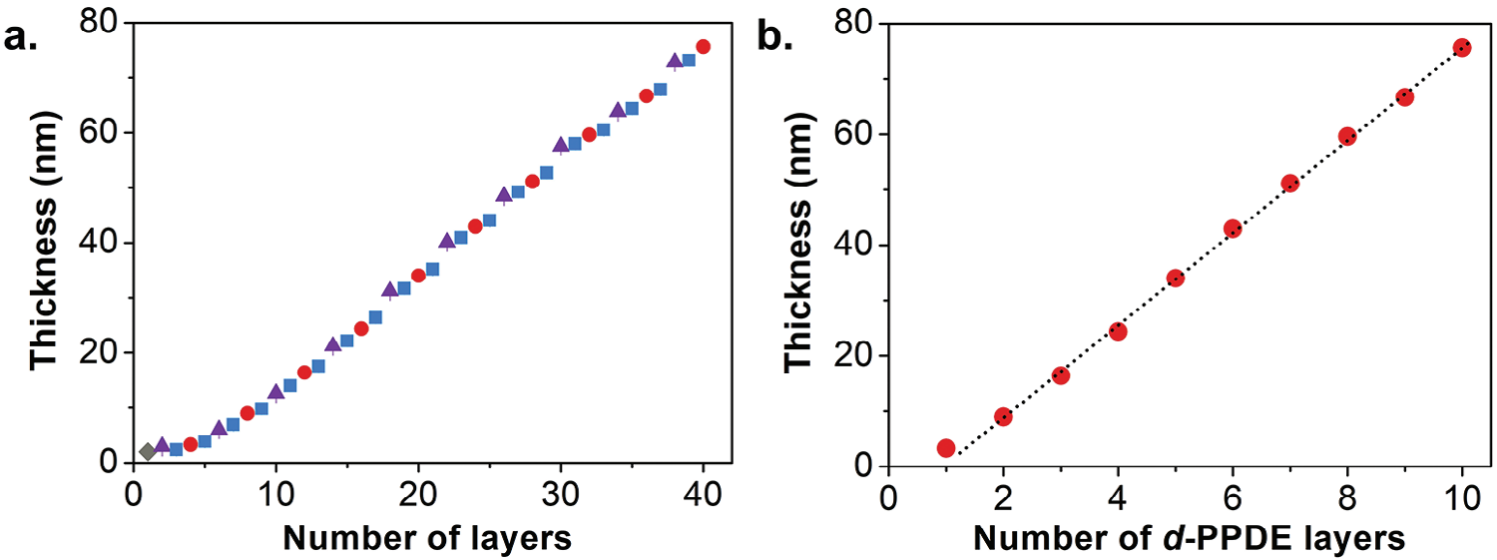
a) Monitoring of film thickness by ellipsometry after deposition of each polyelectrolyte layer in Route 2. In this approach, a 4‐step PSS/PAH/*d*‐PPDE/PAH deposition cycle was followed to deposit 40 consecutive layers. The grey diamond, purple triangles, red circles, and blue squares represent PEI, PSS, *d*‐PPDE, and PAH layers, respectively. b) Monitoring of film thickness by ellipsometry after deposition of each coded *d*‐PPDE layer. The dashed line represents a linear fit showing that the growth of the initial layers is influenced by the substrate and is not representative of that of the bulk film.^[^ ^38^ ^]^

Nevertheless, the average interlayer distance between two coded strata remains relatively small (i.e., less than a nanometer) when Route 1 is used. Due to surface roughness, it may result in layer interpenetration as already known from (PSS/PAH)_n_ films.^[^ ^24^, ^39^, ^40^, ^41^ ^]^ In order to bypass this issue, a second approach was investigated (route 2 in Figure 1b). In this second deposition strategy, PSS was used as a complementary polyanion in order to incorporate a non‐coded PSS/PAH interlayer in between two *d*‐PPDE coded layers. PSS was selected as a co‐polyanion because PSS/PAH is a well‐described polyelectrolyte pair leading to regular and homogenous growth in LbL assembly. This polyelectrolyte pair is known to prevent the diffusion of polyelectrolytes from exponentially growing films.^[^ ^42^ ^]^ In Route 2, a four‐step PSS/PAH/*d*‐PPDE/PAH deposition cycle was followed to deposit 40 consecutive layers including 10 coded *d*‐PPDE layers (**Figure** **2**). After completing the ten deposition cycles, linear growth was observed with a final film thickness of 75 nm and an RMS roughness of ≈0.25 nm (Figure 2b). The interdistance between two *d*‐PPDE coded layers is ≈2–3 nm in these materials, which already seems sufficient for achieving efficient digital segregation but can, of course, further be enlarged if needed by incorporating more number PSS/PAH non‐coded interlayers.

## 3 Conclusion

In summary, a complex *d*‐PPDE heteropolymer was synthesized and evaluated for LbL assembly. Two different routes were compared for constructing multilayer digital films: the direct alternating deposition of PAH and *d*‐PPDE and a four step cycle involving PSS/PAH/*d*‐PPDE/PAH deposition. Ellipsometry monitoring of the LbL process indicated regular and homogenous growth in both strategies. However, the use of non‐coded PSS/PAH intermediate layers seems crucial to efficiently segregate digital layers, a prerequisite for selective reading of the stored digital information. Overall, these results indicate that complex heteropolymers composed of 6 different motifs (i.e., 2 coded monomers, 1 spacer, and 3 mass tags) still enable a finely controlled LbL deposition, despite their marked sequence heterogeneity. Furthermore, the use of polyanions and polycations of markedly‐different molecular weights does not affect film growth. This proof‐of‐principle opens up exciting opportunities for the development of nanostructured data storage materials.

## Conflict of Interest

The authors declare no conflict of interest.

## Supporting information

Supporting Information

## Data Availability

The data that support the findings of this study are available from the corresponding author upon reasonable request.

## References

[1] J.‐F. Lutz , M. Ouchi , D. R. Liu , M. Sawamoto , Science 2013, 341, 1238149. 23929982 10.1126/science.1238149

[2] H. Colquhoun , J.‐F. Lutz , Nat. Chem. 2014, 6, 455. 24848219 10.1038/nchem.1958

[3] M. G. T. A. Rutten , F. W. Vaandrager , J. A. A. W. Elemans , R. J. M. Nolte , Nat. Rev. Chem. 2018, 2, 365.

[4] J.‐F. Lutz , Macromolecules 2015, 48, 4759. 10.1021/ma502518p PMC4822511 27065493

[5] J.‐F. Lutz , ACS Macro Lett. 2014, 3, 1020. 25419485 10.1021/mz5004823 PMC4235389

[6] V. Zhirnov , R. M. Zadegan , G. S. Sandhu , G. M. Church , W. L. Hughes , Nat. Mater. 2016, 15, 366. 27005909 10.1038/nmat4594 PMC6361517

[7] L. Ceze , J. Nivala , K. Strauss , Nat. Rev. Genet. 2019, 20, 456. 31068682 10.1038/s41576-019-0125-3

[8] R. K. Roy , A. Meszynska , C. Laure , L. Charles , C. Verchin , J.‐F. Lutz , Nat. Commun. 2015, 6, 7237. 26006165 10.1038/ncomms8237 PMC4455100

[9] A. C. Boukis , M. A. R. Meier , Eur. Polym. J. 2018, 104, 32.

[10] S. Martens , A. Landuyt , P. Espeel , B. Devreese , P. Dawyndt , F. D.u Prez , Nat. Commun. 2018, 9, 4451. 30367037 10.1038/s41467-018-06926-3 PMC6203848

[11] Z. Huang , Q. Shi , J. Guo , F. Meng , Y. Zhang , Y. Lu , Z. Qian , X. Li , N. Zhou , Z. Zhang , X. Zhu , Nat. Commun. 2019, 10, 1918. 31015480 10.1038/s41467-019-09957-6 PMC6478934

[12] J. M. Lee , M. B. Koo , S. W. Lee , H. Lee , J. Kwon , Y. H. Shim , S. Y. Kim , K. T. Kim , Nat. Commun. 2020, 11, 56. 31911612 10.1038/s41467-019-13952-2 PMC6946701

[13] T. T. Trinh , L. Oswald , D. Chan‐Seng , J.‐F. Lutz , Macromol. Rapid Commun. 2014, 35, 141. 24338828 10.1002/marc.201300774

[14] L. Yu , B. Chen , Z. Li , Q. Huang , K. He , Y. Su , Z. Han , Y. Zhou , X. Zhu , D. Yan , R. Dong , Chem. Soc. Rev. 2023, 52, 1529. 36786068 10.1039/d2cs01022d

[15] J.‐F. Lutz , Eur. Polym. J. 2023, 199, 112465.

[16] J. Steinkoenig , R. Aksakal , F. D.u Prez , Eur. Polym. J. 2019, 120, 109260.

[17] C. Laure , D. Karamessini , O. Milenkovic , L. Charles , J.‐F. Lutz , Angew. Chem., Int. Ed. 2016, 55, 10722. 10.1002/anie.201605279 27484303

[18] M. Frölich , D. Hofheinz , M. A. R. Meier , Commun. Chem. 2020, 3, 184. 36703345 10.1038/s42004-020-00431-9 PMC9814948

[19] Q. Shi , T. Miao , Y. Liu , L. Hu , H. Yang , H. Shen , M. Piao , Z. Huang , Z. Zhang , Macromol. Rapid Commun. 2022, 43, 2200029. 10.1002/marc.202200029 35322486

[20] J. M. Lee , H. Jang , S. W. Lee , K. T. Kim , JACS Au. 2022, 2, 2108. 36186555 10.1021/jacsau.2c00388 PMC9516704

[21] B. É. Petit , B. Lotz , J.‐F. Lutz , ACS Macro Lett. 2019, 8, 779. 35619507 10.1021/acsmacrolett.9b00307

[22] M. Nerantzaki , C. Husser , M. Ryckelynck , J.‐F. Lutz , J. Am. Chem. Soc. 2024, 146, 6456. 38286022 10.1021/jacs.3c13953

[23] R. Szweda , M. Tschopp , O. Felix , G. Decher , J.‐F. Lutz , Angew. Chem. Int. Ed. 2018, 57, 15817. 10.1002/anie.201810559 30290053

[24] G. Decher , Science 1997, 277, 1232.

[25] J. B. Schlenoff , G. Decher , in Multilayer Thin Films: Sequential Assembly of Nanocomposite Materials, 2nd ed., Wiley VCH, Weinheim 2012.

[26] K. Ariga , Y. Lvov , G. Decher , Phys. Chem. Chem. Phys. 2022, 24, 4097. 34942636 10.1039/d1cp04669a

[27] L. Charles , J.‐F. Lutz , Acc. Chem. Res. 2021, 54, 1791. 33729749 10.1021/acs.accounts.1c00038

[28] A. Al Ouahabi , J.‐A. Amalian , L. Charles , J.‐F. Lutz , Nat. Commun. 2017, 8, 967. 29042552 10.1038/s41467-017-01104-3 PMC5645402

[29] K. Launay , J.‐A. Amalian , E. Laurent , L. Oswald , A. Al Ouahabi , A. Burel , F. Dufour , C. Carapito , J.‐L. Clément , J.‐F. Lutz , L. Charles , D. Gigmes , Angew. Chem. Int. Ed. 2021, 60, 917. 10.1002/anie.202010171 32964618

[30] E. Laurent , J. A. Amalian , T. Schutz , K. Launay , J.‐L. Clément , D. Gigmes , A. Burel , C. Carapito , L. Charles , M.‐A. Delsuc , J. F. Lutz , C. R. Chim. 2021, 24, 69.

[31] T. Schutz , I. Sergent , G. Obeid , L. Oswald , A. Al Ouahabi , P. N. W. Baxter , J.‐L. Clément , D. Gigmes , L. Charles , J.‐F. Lutz , Angew. Chem. Int. Ed. 2023, 62, e202310801. 10.1002/anie.202310801 37738223

[32] A. vander Straeten , D. Lefèvre , S. Demoustier‐Champagne , C. Dupont‐Gillain , Adv. Colloid Interface Sci. 2020, 280, 102161. 32416541 10.1016/j.cis.2020.102161

[33] A. Al Ouahabi , L. Charles , J.‐F. Lutz , J. Am. Chem. Soc. 2015, 137, 5629. 25851514 10.1021/jacs.5b02639

[34] A. Al Ouahabi , M. Kotera , L. Charles , J.‐F. Lutz , ACS Macro Lett. 2015, 4, 1077. 35614807 10.1021/acsmacrolett.5b00606

[35] J. A. Amalian , A. Al Ouahabi , G. Cavallo , N. F. König , S. Poyer , J. F. Lutz , L. Charles , J. Mass Spectrom. 2017, 52, 788. 28482377 10.1002/jms.3947

[36] N. Cini , T. Tulun , G. Decher , V. Ball , J. Am. Chem. Soc. 2010, 132, 8264. 20518535 10.1021/ja102611q

[37] C. Picart , P. Lavalle , P. Hubert , F. J. G. Cuisinier , G. Decher , P. Schaaf , J. C. Voegel , Langmuir 2001, 17, 7414.

[38] R. A. McAloney , M. Sinyor , V. Dudnik , M. C. Goh , Langmuir 2001, 17, 6655.

[39] G. Decher , M. Eckle , J. Schmitt , B. Struth , Curr. Opin. Colloid Interface Sci. 1998, 3, 32.

[40] F. Olivier , Z. Zhiqiang , C. Fabrice , D. Gero , C. R. Chim. 2009, 12, 225.

[41] P. Gutfreund , C. Higy , G. Fragneto , M. Tschopp , O. Felix , G. Decher , Nat. Commun. 2023, 14, 4076. 37429844 10.1038/s41467-023-39801-x PMC10333193

[42] F. Boulmedais , M. Bozonnet , P. Schwinté , J. C. Voegel , P. Schaaf , Langmuir 2003, 19, 9873.

