# Supplementary material for: Complex Sequence‐Defined Heteropolymers Enable Controlled Film Growth in Layer‐By‐Layer Assembly
Source: Macromol Rapid Commun. 2024 Aug 6;45(22):2400482. doi: 10.1002/marc.202400482 (PMC11583292; doi:10.1002/marc.202400482)
Supplement: Supplementary file 1 — Supporting Information [file MARC-45-2400482-s001.pdf]

Macromolecular Rapid Communications

Supporting Information

for *Macromol. Rapid Commun.*, DOI 10.1002/marc.202400482

Complex Sequence-Defined Heteropolymers Enable Controlled Film Growth in Layer-By-Layer Assembly

Ranajit Barman, Michel Tschopp, Laurence Charles, Gero Decher, Olivier Felix and Jean-François Lutz**

Supporting Information

Complex sequence-defined heteropolymers enable controlled film growth in layer-by-layer assembly

Ranajit Barman, Michel Tschopp, Laurence Charles, Gero Decher, Olivier Felix and Jean-François Lutz**

A. Experimental Section

A.1. Materials and reagents

Trimethylamine (TEA, >97%, Sigma Aldrich), acetic acid (glacial, Fisher Chemical) trichloroacetic acid (TCA, 99%, Sigma Aldrich), methylamine solution (40% in H_2O, Fluka), aqueous ammonia (30 w% in H_2O, VWR), acetonitrile (ACN, HPLC grade, ≥ 99.93%, Sigma Aldrich) were used as received. Digital poly(phosphodiester)s were synthesized on an automated ABI 3900 DNA Synthesiser (Applied Biosystems) using anhydrous acetonitrile (phosphoramidite diluent & dry washings, ChemGenes), activation reagent (0.25 M 5-ethylthio tetrazole in MeCN, ChemGenes), Cap A (acetic anhydride/pyridine/THF, ChemGenes), Cap B (10 % *N*-methylimidazole in THF, ChemGenes), deprotection reagent (3 w% TCA in dichloromethane, Roth), drying traps (small, 10-15 ml, ChemGenes), and oxidizer (0.02 M iodine/pyridine/H_2O/THF, ChemGenes). The phosphoramidite monomers **0** and **1** and cleavable spacer RISC2 were synthesized as previously described.[1-2] Nucleoside-functionalized column (T-lcaa-CPG, 1000 Å, 1.0 µm) and nucleoside phosphoramidite byte tags (dA-CE phosphoramidite, Ac-dC-CE phosphoramidite) were purchased from Glen Research. Poly(sodium 4-styrenesulfonate) (PSS, *Mw* ~ 70 000 g.mol^{-1}, Sigma Aldrich), poly(allylamine hydrochloride) (PAH, *Mw* ~ 50 000 g.mol^{-1}, Sigma Aldrich), poly(ethylenimine) (PEI, Lupasol WF, *Mw* ~ 25 000 g.mol^{-1}, BASF) were used in LbL studies.

A.2. Automated synthesis of digital poly(phosphodiester)s

The sequence-defined polymer studied herein was synthesized by automated phosphoramidite chemistry, using previously-reported protocols on an automated 3900 DNA synthesiser (Applied Biosystems).[3-4] The solutions of phosphoramidite monomers **0** and **1**, spacer RISC2 and byte tags nucleoside phosphoramidite (dA-CE, Ac-dC-CE) were prepared in anhydrous ACN (0.06 M) in the bottles of the synthesizer. Thymidine-loaded ABI 3900 HT T columns (1 µmol in cartridge, Applied Biosystems) were utilized to synthesize the polymers in DMT-on

mode, in which the terminal DMT protective unit will remain intact. After completion of polymer synthesis, the column was removed from the synthesizer and the DMT protected polymer was cleaved from the solid support using a mixture solution (1:1 v/v) of $MeNH_2$ (40% in water) and NH_3 (30% in water) for 30-45 min at room temperature. Then the terminal DMT group of the polymer was removed and the desired sequence coded polymer was purified using reverse-phase columns (Glen-Pak, DNA purification cartridge, Glen Research) and washed out by solvent elution. The aqueous polymer solution was lyophilized to obtain the polymer as a white solid powder.

A.3. Thin films preparation by LbL deposition

The layer-by-layer (LbL) deposition of the sequence encoded *d*-PPDE was done on silicon wafers (Si 100, WaferNet Inc.) by manual dipping method. Firstly, silicon wafers were cut in 10 × 20 mm dimension and washed with diluted alkaline solution and then ultrasound sonication was performed for 15 min in a mixture solution (1:1 v/v) of ethanol and Milli-Q water and dried by compressed air. Then plasma cleaning was carried out by keeping them in high intensity plasma dose for 2-3 min using a plasma cleaner (Harrick Plasma). All polyelectrolytes were dissolved in volumetric flasks in 0.15M NaCl solution of Milli-Q water at the following concentrations: PSS (0.06%), PAH (0.03%) and *d*-PPDE (0.02%). All volumetric flasks were washed properly with a 2 % (v/v) Hellmanex (Hellma GmbH, Germany) and Milli-Q water before use. The plasma-activated substrates were immersed manually in PEI solution (0.25%) for 15 min, washed 3 times by 2 min dipping in Milli-Q water and dried by compressed air. Each PEI functionalized substrate was dipped successively into polyanion and polycation solutions following two different routes (schematized in Figure 1c of the main text). The composition of the formed film was PEI-$(d\text{-PPDE/PAH})_n$ for route-1 and PEI-$(\text{PSS/PAH/}d\text{-PPDE/PAH})_n$ for route-2. After each layer deposition, the thickness of the film was measured by ellipsometry.

B. Measurements and analysis

B.1. Ion exchange High Pressure Liquid Chromatography (HPLC)

Ion exchange HPLC (IE-HPLC) analysis was performed using an Agilent 1220 Infinity II LC System with a dual channel gradient pump (with degasser) and a UV detector (260 and 280 nm). All samples passed through an ion-exchange column (DIONEX, DNA Pac PA100, 4 x 250 mm) and the chromatograms were recorded at λ=260 nm. Elution was performed applying a linear gradient of Phase B (from 5 to 30%) over 27 min at a flow rate of 1.0 mL/min. Phase A was

prepared with 10% acetonitrile and 20% NH_3 (2 M) in milli-Q water and Phase B with 2.5 M NaCl in milli-Q water. The raw IE–HPLC data were processed using Origin 2020.

B.2. Ellipsometry

The thickness measurements were performed with a PLASMOS SD 2300 operating at a wavelength of 632.8 nm at an incidence angle of 70° at RT. For thickness measurements, the refractive index was set at $n = 1.465$ and assumed to be constant. Each data point is an average of 10 measurements at random positions on the wafer. This procedure leads to slightly inexact absolute thickness values, but it allows the determination of the thickness and sufficient precision for the comparison of the buildup and homogeneity of the different films.

B.3. Mass spectrometry

High resolution mass spectrometry (MS) experiments were performed with a Synapt G2 HDMS instrument from Waters (Manchester, UK), operated in the negative ion mode (capillary ESI voltage, –2.27 kV; sampling cone voltage, –20 V; extraction cone voltage, –6 V). The desolvation gas (N_2) flow was 100 L/h at 35°C. A syringe pump was used to introduce sample solutions in the ESI source at a 10 µL/min flow rate. In MS/MS experiments, deprotonated *d*-PPDEs were selected in the Q1 quadrupole and activated in the collision cell to release blocks. For pseudo-MS^3 sequencing, the cone voltage was changed to –80 V in order to induce in-source fragmentation of deprotonated *d*-PPDEs and generate blocks in the interface of the instrument; then, each block was mass-selected in the Q1 quadrupole and activated in the collision cell for sequencing. All activation stages used argon as the collision gas. Instrument control, data acquisition and data processing of all experiments were achieved with the MassLynx 4.1 programs provided by Waters.

C. Additional Figures:

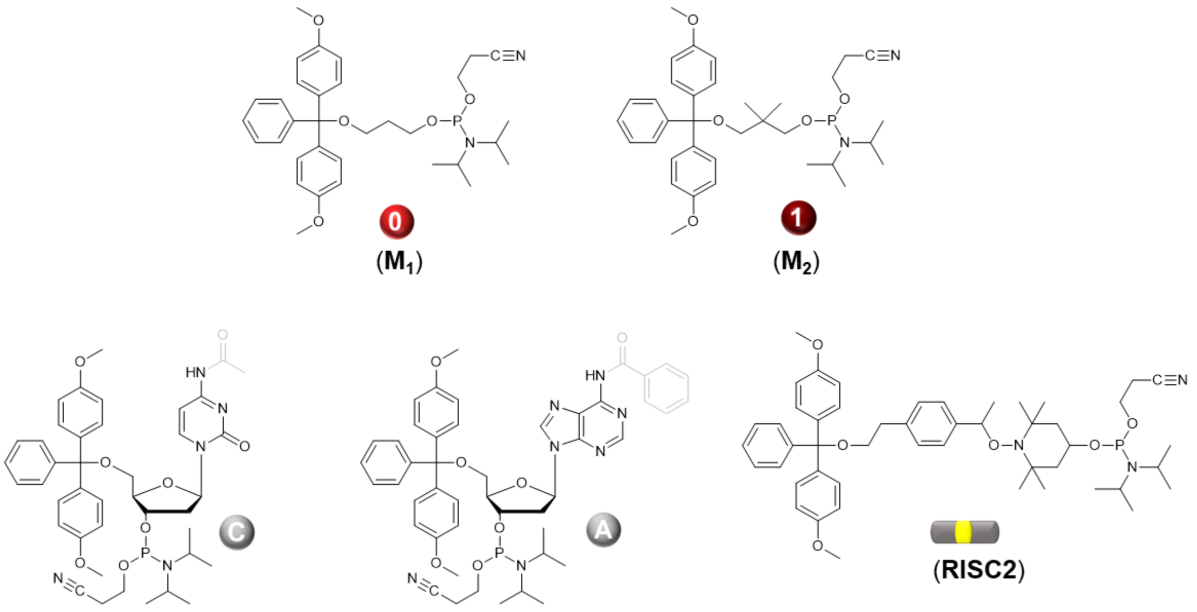

Figure S1. Molecular structure of the phosphoramidite monomers used in this work for synthesizing the *d*-PPDE heteropolymer.

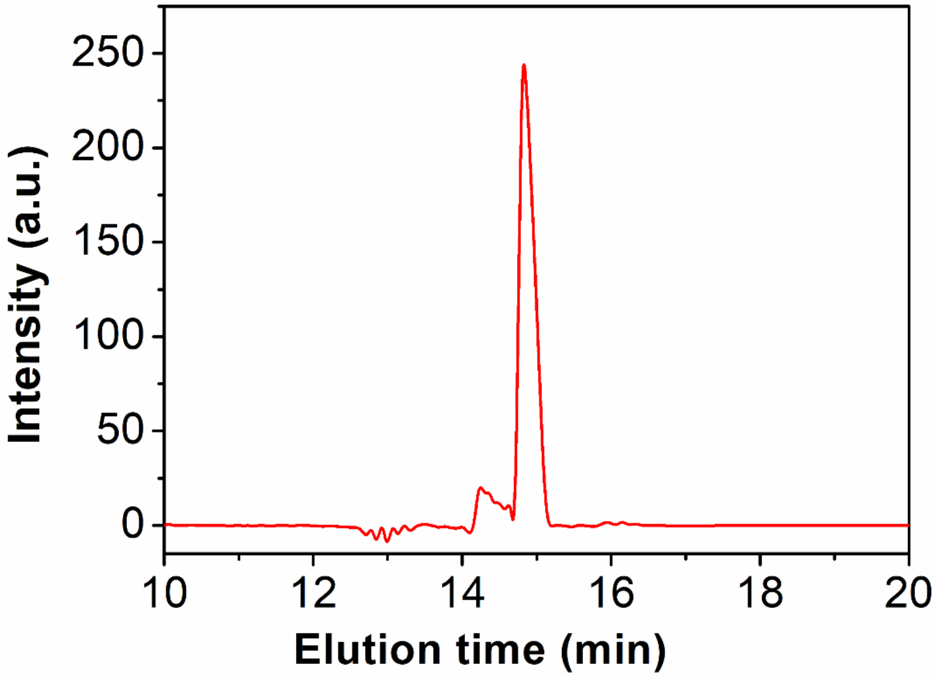

Figure S2. IE-HPLC chromatogram recorded for the *d*-PPDE heteropolymer.

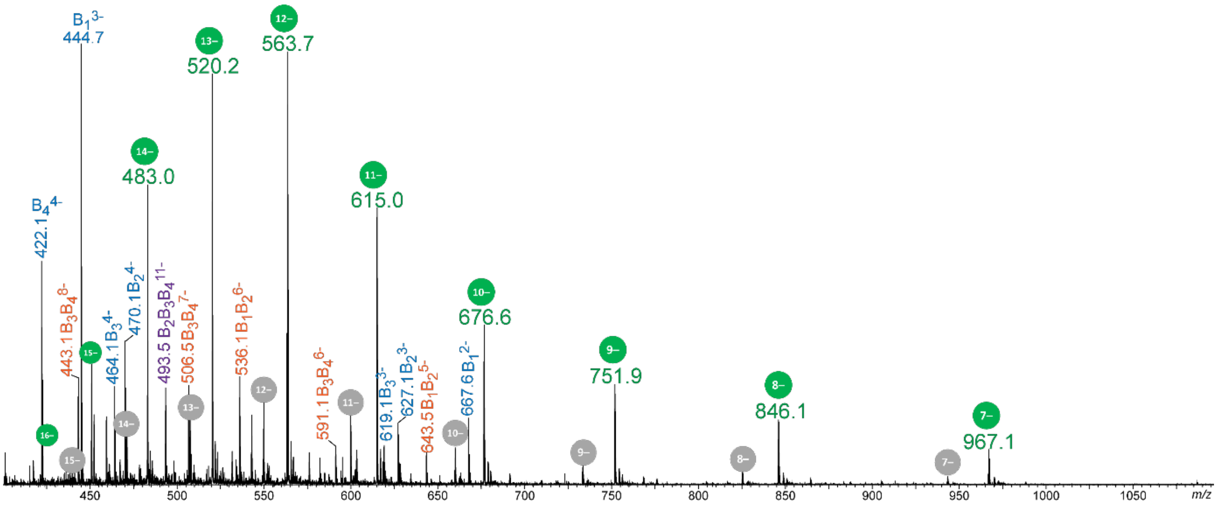

Figure S3. ESI(–)-MS of the *d*-PPDE sample, with peaks of the targeted sequence annotated with their *m/z* and charge state (in green) and signals of the minor impurity lacking one 1-bit (Δm –166 Da) annotated in grey. In-source cleavage of alkoxyamine bonds in the main chains lead to the release of fragments comprised of one block (B_i^{z-}, in blue), two blocks ($B_iB_j^{z-}$, in orange) or three blocks ($B_iB_jB_k^{z-}$, in purple).

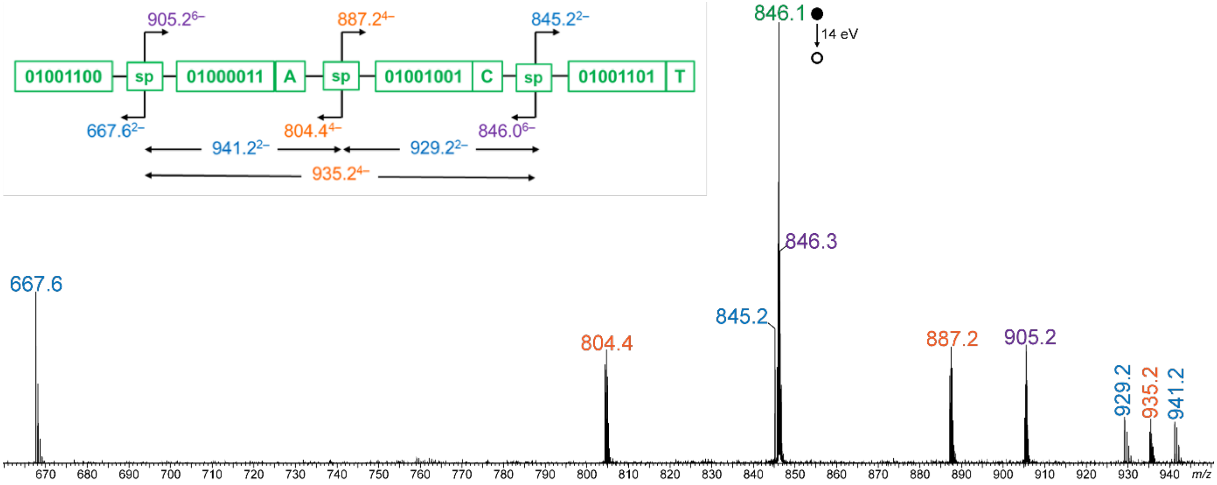

Figure S4. MS/MS of [*d*-PPDE – 8H]$^{8-}$ at *m/z* 846.1, with specific cleavage of alkoxyamine bond leading to product ions that enable reconstruction of the original sequence of blocks as depicted by the inset scheme.

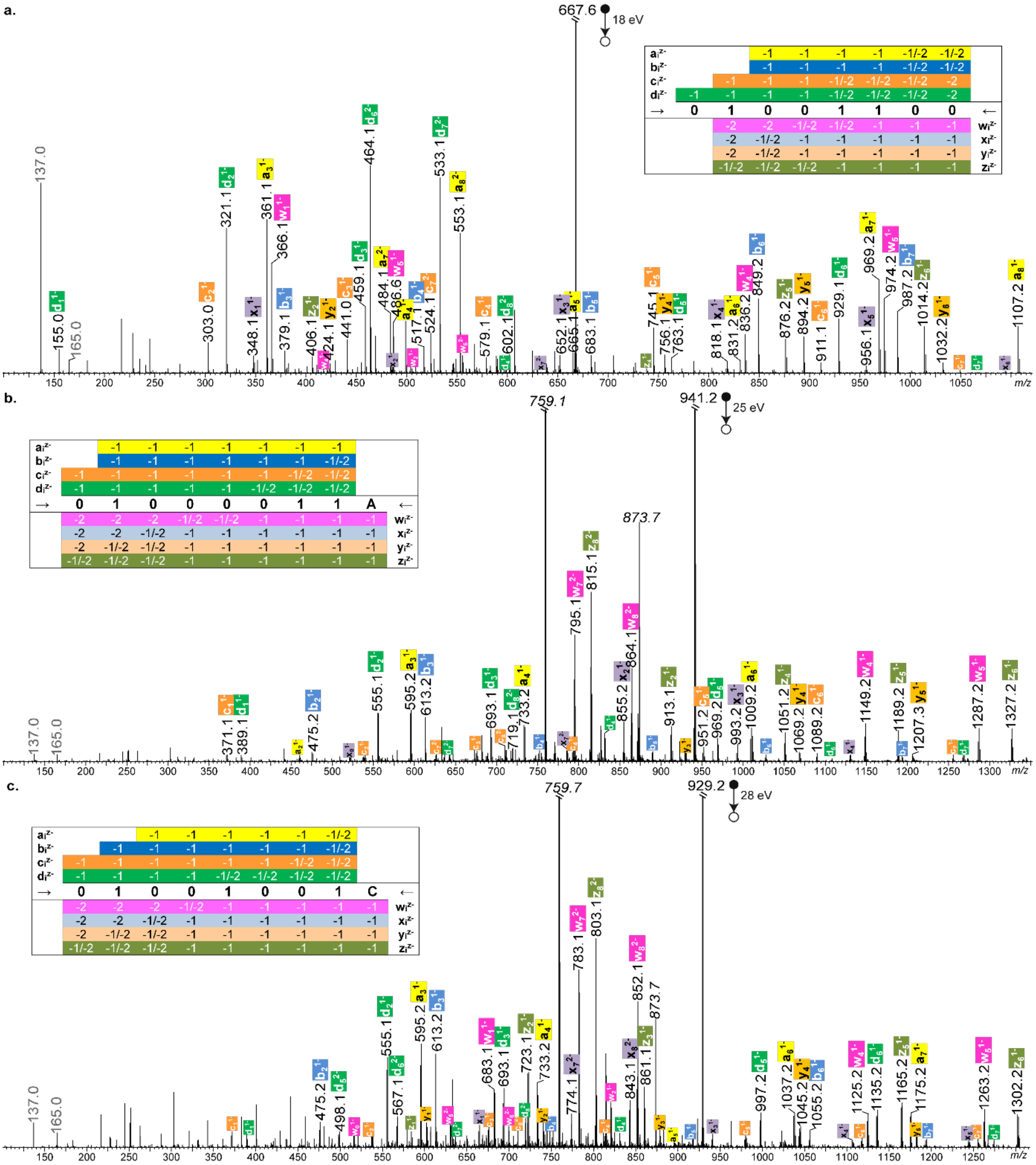

Figure S5. Pseudo-MS^3 sequencing of individual blocks with sequence coverage in inset tables. (**a**) [B1 – 2H]$^{2-}$ at *m/z* 667.6, with 01001100 sequence coding for “L”, (**b**) [B2 – 2H]$^{2-}$ at *m/z* 941.2, with 01000011 sequence coding for “C”, and (**c**) [B3 – 2H]$^{2-}$ at *m/z* 929.2, with 01001001 sequence coding for “I” and d) [B4 – 2H]$^{2-}$ at *m/z* 845.2, with 01001101 sequence coding for “M”. Deprotonated coding monomers are in grey, namely [0 – H]$^{-}$ at *m/z* 137.0 and [1 – H]$^{-}$ at *m/z* 165.0. Unlabeled peaks correspond to internal fragments. Ions annotated with italicized values in (**b**) and (**c**) are produced by radical-induced dissociation of the nucleoside tag.

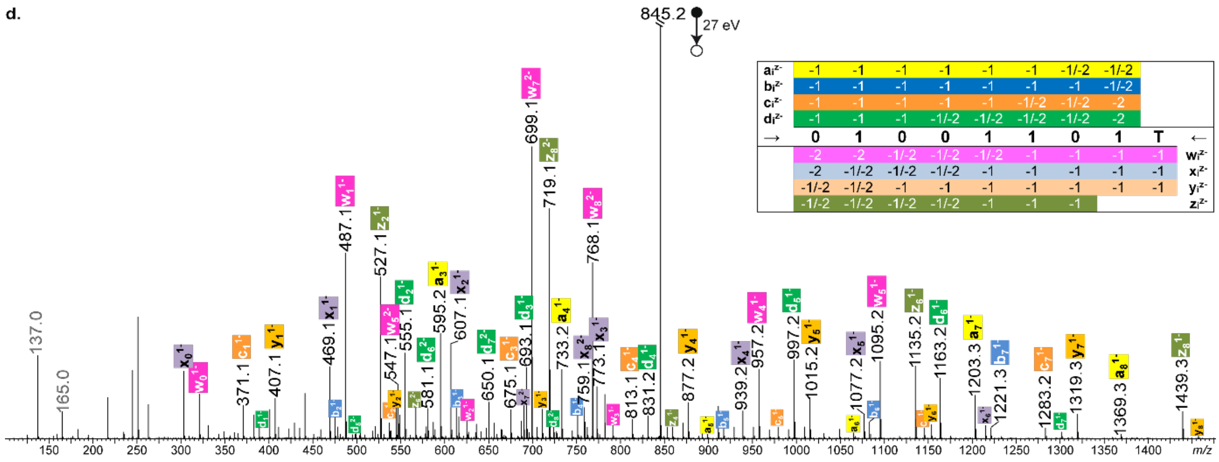

Figure S5. Pseudo-MS^3 sequencing of individual blocks with sequence coverage in inset tables. (**d**) [B4 – 2H]$^{2-}$ at *m/z* 845.2, with 01001101 sequence coding for "M". Deprotonated coding monomers are in grey, namely [0 – H]$^-$ at *m/z* 137.0 and [1 – H]$^-$ at *m/z* 165.0. Unlabeled peaks correspond to internal fragments.

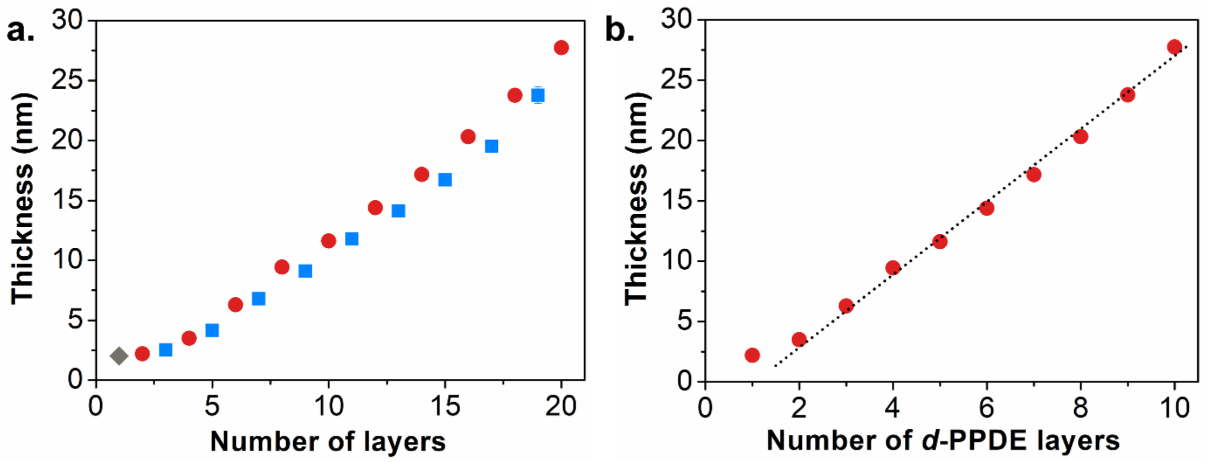

Figure S6. (**a**) Monitoring of film thickness by ellipsometry after deposition of each polyelectrolyte layer in Route 1. In this approach, a 2-step PAH/*d*-PPDE deposition cycle was applied to deposit 20 consecutive layers. The grey diamond, red circles and blue squares represent PEI, *d*-PPDE and PAH layers, respectively. (**b**) Monitoring of film thickness by ellipsometry after deposition of each coded *d*-PPDE layer. The dashed line represents a linear fit showing that the thickness growth of initial layers is influenced by the substrate and is not representative of that of the bulk film.[5]

D. References

[1] A. Al Ouahabi, L. Charles, J.-F. Lutz, *J. Am. Chem. Soc.* **2015**, *137*, 5629-5635.

[2] K. Launay, J.-A. Amalian, E. Laurent, L. Oswald, A. Al Ouahabi, A. Burel, F. Dufour, C. Carapito, J.-L. Clément, J.-F. Lutz, L. Charles, D. Gigmes, *Angew. Chem., Int. Ed.* **2021**, *60*, 917-926.

[3] A. Al Ouahabi, M. Kotera, L. Charles, J.-F. Lutz, *ACS Macro Lett.* **2015**, *4*, 1077-1080.

[4] E. Laurent, J. A. Amalian, T. Schutz, K. Launay, J.-L. Clément, D. Gigmes, A. Burel, C. Carapito, L. Charles, M.-A. Delsuc, J. F. Lutz, *C. R. Chim.* **2021**, *24*, 69-76.

[5] R. A. McAloney, M. Sinyor, V. Dudnik, M. C. Goh, *Langmuir* **2001**, *17*, 6655-6663.
